# Whole genome comparison of donor and cloned dogs

**DOI:** 10.1038/srep02998

**Published:** 2013-10-21

**Authors:** Hak-Min Kim, Yun Sung Cho, Hyunmin Kim, Sungwoong Jho, Bongjun Son, Joung Yoon Choi, Sangsoo Kim, Byeong Chun Lee, Jong Bhak, Goo Jang

**Affiliations:** 1 Personal Genomics Institute, Genome Research Foundation, Suwon 443-270, Korea; 2 Theragen BiO Institute, TheragenEtex, Suwon 443-270, Korea; 3 School of Systems Biomedical Science, Soongsil University, Seoul 156-743, Korea; 4 Department of Theriogenology, College of Veterinary Medicine and the Research Institute of Veterinary Science, Seoul National University, Korea; 5 Program in Nano Science and Technology, Department of Transdisciplinary Studies, Seoul National University, Suwon 443-270, Korea; 6 Advanced Institutes of Convergence Technology Nano Science and Technology, Suwon 443-270, Korea; 7 Emergence Center for Food-Medicine Personalized Therapy System, Advanced Institutes of Convergence Technology, Seoul National University, Gyeonggi-do, Korea; 8 These authors contributed equally to this work.

## Abstract

Cloning is a process that produces genetically identical organisms. However, the genomic degree of genetic resemblance in clones needs to be determined. In this report, the genomes of a cloned dog and its donor were compared. Compared with a human monozygotic twin, the genome of the cloned dog showed little difference from the genome of the nuclear donor dog in terms of single nucleotide variations, chromosomal instability, and telomere lengths. These findings suggest that cloning by somatic cell nuclear transfer produced an almost identical genome. The whole genome sequence data of donor and cloned dogs can provide a resource for further investigations on epigenetic contributions in phenotypic differences.

Dogs are one of the invaluable animal models in biomedical fields, because they exhibit 333 genetic diseases that are similar to human's^1^. In 2005, the clone of a male Afghan hound, named "Snuppy", was generated^2^,^3^ by somatic cell nuclear transfer (SCNT), which is a form of cloning that transfers the nucleus from a somatic cell into an oocyte. Snuppy has grown up without any detectable abnormality to date. He and other cloned dogs also seem to be normally fertile, as artificial insemination with two cloned female dogs resulted in 10 healthy puppies being born in 2009^4^.

Cloned offspring can be exposed to different environments, whereas identical twins usually grow up under very similar conditions right from birth. Therefore, cloning by SCNT is an invaluable model to study the effect of the environment on the phenotype. However, it has not been confirmed that their whole length genomes are indeed identical. Fortunately, the full reference genome of a dog has already been assembled^5^ and is publicly available. Here we carried out whole genome sequencing of the cloned dog and its nuclear donor dog (Supplementary Fig. S1), in order to compare them with the dog assembly. To investigate the level of genomic difference in the dogs, we compared it with the genomes of human monozygotic twins (ethnic Korean, female), which serve as an example of natural cloning and were assumed to be of identical genetic make-up^6^. We carried out a genome-wide analysis in terms of single nucleotide variation (SNV), copy number variation (CNV), structural variation (SV), and telomere lengths (Fig. 1 and Table 1).

## Results

### Whole genome sequences of donor and cloned dogs

The DNA of a male cloned dog (Snuppy, 7.5 years old) and a male donor dog (Tai, 10.5 years old) was sequenced using Illumina HiSeq2000 (Supplementary Table S1 and Methods). On average, 56 gigabases per sample (~20 × depth) were produced (Supplementary Table S2) and were mapped to the dog reference genome (CanFam3.1) at a mapping rate of over 98% (Supplementary Table S3). In both dogs, on average, about 4.4 million SNVs and 1.1 million small insertions and deletions (indels) were identified (Supplementary Tables S4 and S5). When the variations were compared, 8,534 SNVs (8,337 autosomal, 115 sex chromosomal, and 82 mitochondrial) and 6,872 small indels (6,789 autosomal, 82 sex chromosomal, and 1 mitochondrial) from the cloned dog were detected as somatic (i.e., post-cloning *de novo*) variations (Supplementary Table S6). These are comparable to those of the monozygotic twin genomes (9,129 somatic (post-twinning *de novo*) SNVs and 3,509 somatic indels) that have been analyzed by the same methods. Additionally, the mutation rate of the cloned dog (3.77 mutations/Mb) was comparable to those of the donor dog (3.84 mutations/Mb) and twins (3.36–3.57 mutations/Mb) (Supplementary Fig. S2 and Supplementary Table S7). The number of mitochondrial somatic variations of the cloned dog was higher than that of the twin (zero mitochondrial somatic variation), and this was expected as the cloned dog's mitochondrial DNA was transmitted by an oocyte donor. The somatic variation patterns of nucleotide substitution is an important element in disease research, such as cancers^7^, and we found that the variation patterns of the dogs and twins showed a high level of similarity (bias into the transitions of A > G and C > T) (Supplementary Fig. S3). These results suggest that the SCNT did not cause any altering of the mutation rates and patterns.

### Identification of *de novo* mutation in cloned dog

Notably, only six somatic autosomal nonsynonymous SNVs (nsSNVs in *DNAJC14*, *KNTC1*, *ZNF683*, *KAT6B*, *ESCO1*, and ENSCAFG00000030636 genes) were found in the cloned dog. While occurring in different genes, an identical number of nsSNVs was found in the monozygotic twin (*PRB3*, *TMC5*, *DISP1*, *SALL4*, *SPATS1*, and *C9orf139* genes; Supplementary Table S8). Additionally, the cloned dog and twin did not show any insertion or deletion in coding regions. Upon in-depth analyses using computational prediction (PolyPhen2)^8^ among the genes containing nsSNVs, only *ESCO1* (K811E) in the cloned dog and *SPATS1* (G8R) and *C9orf139* (D49N) in the monozygotic twin were predicted to be function altered (probably or possibly damaging). Interestingly, the *ESCO1* gene, which belongs to a conserved family of acetyltransferases, is involved in sister chromatid cohesion in the S phase of the mitotic cell cycle^9^. Also, the *KNTC1* gene has an nsSNV (E1204D, neutral) in the cloned dog, which is known to be an essential component of the mitotic checkpoint and prevents cells from prematurely exiting mitosis in M phase^10^. Although these mutations occurred in the genes that are associated with the cell cycle, all of the somatic nsSNVs were heterozygous variations, perhaps indicating proper function of the genes. Furthermore, there was no experimental evidence that the cloning caused any abnormality in the cell cycle, as the cultured cell lines derived from the donor and cloned dogs grew without any obvious differences.

### Chromosomal instability analysis

Chromosomal instability, such as CNV and SV, is important in disease research^11^. The analysis showed that there was no CNV difference between the donor and cloned dogs, with the exception of three CNV differences in mitochondrial DNA that were caused by a different oocyte. This was fewer than the human twins who had only two CNV differences in the autosome (Supplementary Table S9). This result indicates that the clone had almost identical genomic structure to that of the nuclear donor. Additionally, we found 903 and 778 SV signals from the donor dog and cloned dog, respectively. Among them, only 12 SVs (1.5%) were identified as somatic SVs (Supplementary Table S10). This is much fewer than that of the monozygotic twin (394 somatic SVs, 25.1%). Four out of the 12 somatic SVs in the cloned dog were located in the intron regions of *HPS5*, *AGPS*, and *FAM73A* (insertions) genes, and only one exon region of the unknown gene (ENSCAFG00000015277) suffered from inter-chromosomal translocation (Supplementary Table S11). On the other hand, 116 of the twin's genes were affected by the somatic SVs. In short, these chromosomal instability analyses revealed that the degree of similarity in the cloned dog is higher than that of the twin, especially when considering the age effect as the human equivalent biological age of the dogs was higher than the twins' age (40 to 70 years compared with 20 years, respectively).

### Telomere length of donor and cloned dogs

Telomeres protect the ends of chromosomes and are reduced in length in most mammalian cell types during replication^12^. When telomeres reach a critically short length, a DNA damage signal is initiated, inducing cell senescence^13^. Telomere length is one of the major issues in cloned offspring; while the first cloned sheep, Dolly, had a significantly shorter telomere than that of an age-matched control^14^, the lengths of cloned cattle and mice showed the same or longer telomeres than those of the normal calves^15^,^16^. Moreover, previous reports suggest that telomere length correlates with the life span of dog breeds^13^. Therefore, we estimated the telomere lengths of the donor and cloned dog using whole genome sequencing data^17^ (see Methods). Interestingly, the estimated relative telomere lengths of the two dogs were very similar (Supplementary Table S12). A previous experimental examination, which was performed when the cloned dog was one year old (Supplementary Fig. S4), showed the same result. This result coincides with the phenotypic observation that the cloned dog and his offspring are healthy and show no early signs of senescence (Supplementary Fig. S1). However, it is known that cloned animals tend to have a more compromised immune function and higher rates of infection, tumor growth, and other disorders^18^,^19^. Therefore, there may well be epigenetic factors affecting the health of cloned animals in general.

## Discussion

We report the genome-wide analyses of a cloned dog, which is, to the best of our knowledge, the first whole genome sequenced from cloned animals. The donor and cloned dogs showed a high level of genome similarity, comparable with the genomes of human monozygotic twins. Genetically identical individuals can be used to study disease mechanisms and therapies^20^. Additionally, they provide an invaluable resource for investigating epigenetic and environmental contributions to the diverse biological and behavioral traits associated with the many different canine breeds^21^,^22^,^23^.

## Methods

### Sample preparation and whole genome sequencing

Genomic DNA was extracted from blood collected from the jugular vein of both the cloned and original donor dogs from Seoul National University of Korea with the PAXgene Blood DNA Kit (Qiagen, Valencia, CA, USA), following the manufacturer's protocol. The human monozygotic twins' DNA came from the Korean Personal Genome Project (KPGP, available at http://opengenome.net). A library of ~ 280 bp insert size was constructed at Theragen BiO Institute (TBI), TheragenEtex, Korea. Genomic DNA was sheared using Covaris S series (Covaris, MS, USA). The sheared DNA was end-repaired, A-tailed, and ligated to paired-end adapters, according to the manufacturer's protocol (Truseq DNA Sample Prep Kit v2, Illumina, San Diego, CA, USA). Adapter-ligated fragments were then size selected on a 2% Agarose gel, with the 400–500 bp band being extracted. Gel extraction and column purification process was performed using the Minelute Gel Extraction Kit (Qiagen), following the manufacturer's protocol. The ligated DNA fragments which contained adapter sequences were enhanced via PCR using adapter specific primers. Library quality and concentration were determined using an Agilent 2100 BioAnalyzer (Agilent). The libraries were quantified using a SYBR green qPCR protocol on a LightCycler 480 (Roche, Indianapolis, IN, USA), according to Illumina's library quantification protocol. Based on the qPCR quantification, the libraries were normalized to 2 nM and then denatured using 0.1 N NaOH. Cluster amplification of denatured templates was performed in flow cells, according to the manufacturer's protocol (Illumina). Flow cells were paired-end sequenced (2 × 100 bp) on an Illumina HiSeq2000 using HiSeq Sequencing kits. A base-calling pipeline (Sequencing Control Software (SCS), Illumina) was used to process the raw fluorescent images and the called sequences.

### Raw read filtering

For the genome-wide analysis, the raw read sequences of the donor dog and cloned dog and the monozygotic twins were filtered using following criteria: 1) Reads with ambiguous bases (represented by the letter N) exceeds 10%. 2) Average quality of the read is under 15. 3) Nucleotides under quality 15 exceed 10% of a read. 4) For any read which contains an adapter sequence: A. More than 10 bp of the tail of the first read and the head of the index adapter are identical. B. More than 10 bp of the tail of the second read and the head of the universal adapter complementary sequence are identical. Finally, the rmdup command of SAMtools^24^ was used to remove PCR duplicates of sequence reads, which can be generated during the library construction process.

### Read alignment and variation (SNVs or indels) detection

Paired-end sequence reads were aligned to the dog (CanFam3.1) and human (hg19) reference genomes with the BWA^25^ ver. 0.5.9. Two mismatches were permitted in a 45 bp seed sequence. Aligned reads were realigned at putative indel positions with the Genome Analysis Toolkit (GATK)^26^ IndelRealigner algorithm to enhance the mapping quality. Base quality scores were recalibrated using the TableRecalibration algorithm of GATK. Putative SNVs were called and filtered using the UnifiedGenotyper and VariantFiltration commands in GATK. The options used for SNV calling were a read mapping depth of 5–200 with a consensus quality of 10 and a prior likelihood for heterozygosity value of 0.001. To obtain small indels, the Unified Genotyper DINDEL mode of GATK was used with default values, including a window size of 300.

### Somatic variation detection and filtering

To identify somatic variations, variations from the cloned dog genome were filtered using the variations from the donor dog genome using VarScan^27^ ver. 2.3.4 with default options. In the same manner, the somatic variations of monozygotic twins were identified by filtering variations from one twin genome by the mutations from the other twin genome. The somatic variations with *P* > 0.05 were filtered out. All somatic variations altering amino acid sequences were checked by expert lab personnel using the tview command of SAMtools. SnpEff^28^ was used to annotate the variations.

### Mutation rate calculation

For the mutation rate calculation, the number of SNVs was compared to the total number of bases in sufficiently covered region. The sufficiently covered region was defined where its read mapping depth is between 5 and 200 reads.

### Identification of copy number variations (CNVs) and structural variations (SVs)

CNVs based on the differences in sequencing depths between the two dog genomes and monozygotic twin genomes were detected using BIC-seq^29^ v1.1.2 with λ = 2, bin_size = 100 bp, multiplicity = 2, window = 200, insert_size = 265 (sd:20), and paired options. As the input of the BIC-seq, the cloned dog and donor dog were considered as case and control cases, respectively. Regions with a log2 ratio smaller than −0.2 or larger than 0.2 were defined as deleted or duplicated regions, respectively. SVs were scanned using BreakDancer^30^ with the score > = 80, size > = 1000 and read coverage > = 10 were used with cloned dog or monozygotic twins, respectively. To identify somatic SVs, the SVs of the cloned dog were filtered out using the SVs from the nuclear donor dog genome.

### Telomere length estimation

Relative telomere lengths of the cloned dog and donor dog were estimated by dividing the number of reads having ‘TTAGGG’ repeat (from 1 to 6 repeats) by the number of total reads as described in a previous report^17^. To normalize bias from sequencing quality, other repeats, such as ‘GGGATT’, were also used as controls. Southern blotting is also used to validate the telomere lengths in experiments. Mean telomere length was determined by mean terminal restriction fragment (TRF) length analysis with a TeloTAGGG Telomere Length Assay kit (Roche, Mannheim, Germany). The isolated genomic DNA (5 ug) was digested with restriction enzymes, Hinf I and Rsa I (New England Biology) digested genomic DNA samples were fractionated by agarose gel (0.8%) transferred to a positive charge nylon membrane (Hybond +, Amersham Pharmacia Biotech., Oakville, Canada). The membranes were prehybridizied in 40 mL of DIG Eeasy Hyb (Roche) for 2 hrs at 42°C, and then hybridized in 10 ml of DIG Easy Hyb containing 50 pmol of end-labeled, telomere-specific probe for 16 hrs at 42°C. Membranes were washed three times in 50 ml of 0.5 × standard saline citrate (SSC; 1 × SSC; 0.15 M NaCl, 0.015 M Sodium Citrate) for 15 mins at room temperature. The signals were visualized by chemiluminescence using a DIG Luminescent Detection Kit (Roche) and exposed by to x-ray film (Hyperfilm, Amersham Pharmacia Biotech.). The signals were scanned and analyzed using Gel Doc software (Bio-rad, Hercules, CA).

## Author Contributions

H.K., H.-M.K., S.J. and Y.S.C. performed the bioinformatic analysis of whole genome sequence data. J.Y.C. performed the genome sequencing. H.-M.K., Y.S.C., H.K., S.K., B.S., G.J. and J.B. wrote the manuscript. J.B., B.C.L. and G.J. conceived and designed this study.

## Additional information

**Accession codes:** Whole genome sequence data of the cloned dog and donor dog used for this analysis are available at Short Read Archive (SRA) under accession code SRP025974.

## Supplementary Material

Supplementary Information Supplementary Information

## Figures and Tables

**Figure 1:**
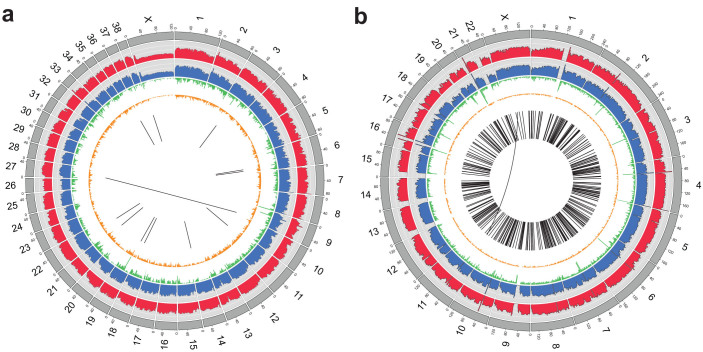
Overview of somatic alterations in cloned dog and monozygotic twin. (a) Difference of alterations in cloned and donor dogs. (b) Difference of alterations in monozygotic twins. From the outside, each layer represents reference chromosomes (grey), mapping depth of cloned and donor dogs (red and blue, respectively), the number of somatic SNVs (green), and the number of somatic indels (yellow). The difference of structural variations (SVs) is shown as black lines in the center (the short-lines indicate insertion, deletion, and intra-chromosomal translocation; the long-lines across the centers indicate inter-chromosomal translocation).

**Table 1:** Global statistics of the cloned dog and monozygotic twin

	Cloned dog	Monozygotic Twin
Year of birth	2005	1990
Avg. mapping depth	>20 ×	>21 ×
# of somatic SNV	8,534	9,129
# of somatic Indel	6,872	3,509
Somatic mutation rate (/Mbase)	3.77	3.57
# of somatic nsSNV	6	6
# of somatic CNV	3 (in mtDNA)	2
# of somatic SV	12	394
