# Supplementary material for: Whole genome comparison of donor and cloned dogs
Source: Sci Rep. 2013 Oct 21;3:2998. doi: 10.1038/srep02998 (PMC3801109; doi:10.1038/srep02998)
Supplement: Supplementary Information [file srep02998-s1.doc]

**Supplementary Information**

**Whole genome comparison of donor and cloned dogs**

Hak-Min Kim^1,‡^, Yun Sung Cho^1,‡^, Hyunmin Kim^2,‡^, Sungwoong Jho^1^, Bongjun Son^2^, Joung Yoon Choi^2^, Sangsoo Kim^3^, Byeong Chun Lee^4^, Jong Bhak^1,2,5,6,*^ & Goo Jang^4,7,*^

^1^Personal Genomics Institute, Genome Research Foundation, Suwon 443-270, Republic of Korea

^2^Theragen BiO Institute, TheragenEtex, Suwon 443-270, Republic of Korea

^3^School of Systems Biomedical Science, Soongsil University, Seoul 156-743, Republic of Korea

^4^Department of Theriogenology, College of Veterinary Medicine and the Research Institute of Veterinary Science, Seoul National University, Republic of Korea

^5^Program in Nano Science and Technology, Department of Transdisciplinary Studies, Seoul National University, Suwon 443-270, Republic of Korea

^6^Advanced Institutes of Convergence Technology Nano Science and Technology, Suwon 443-270, Republic of Korea

^7^Emergence Center for Food-Medicine Personalized Therapy System, Advanced Institutes of Convergence Technology, Seoul National University, Gyeonggi-do, Korea

^*^Correspondence and requests for materials should be addressed to G.J. (snujang@snu.ac.kr) or J.B. (jongbhak@genomics.org)

^‡^These authors contributed equally to this work.

*Supplementary information figures*

**
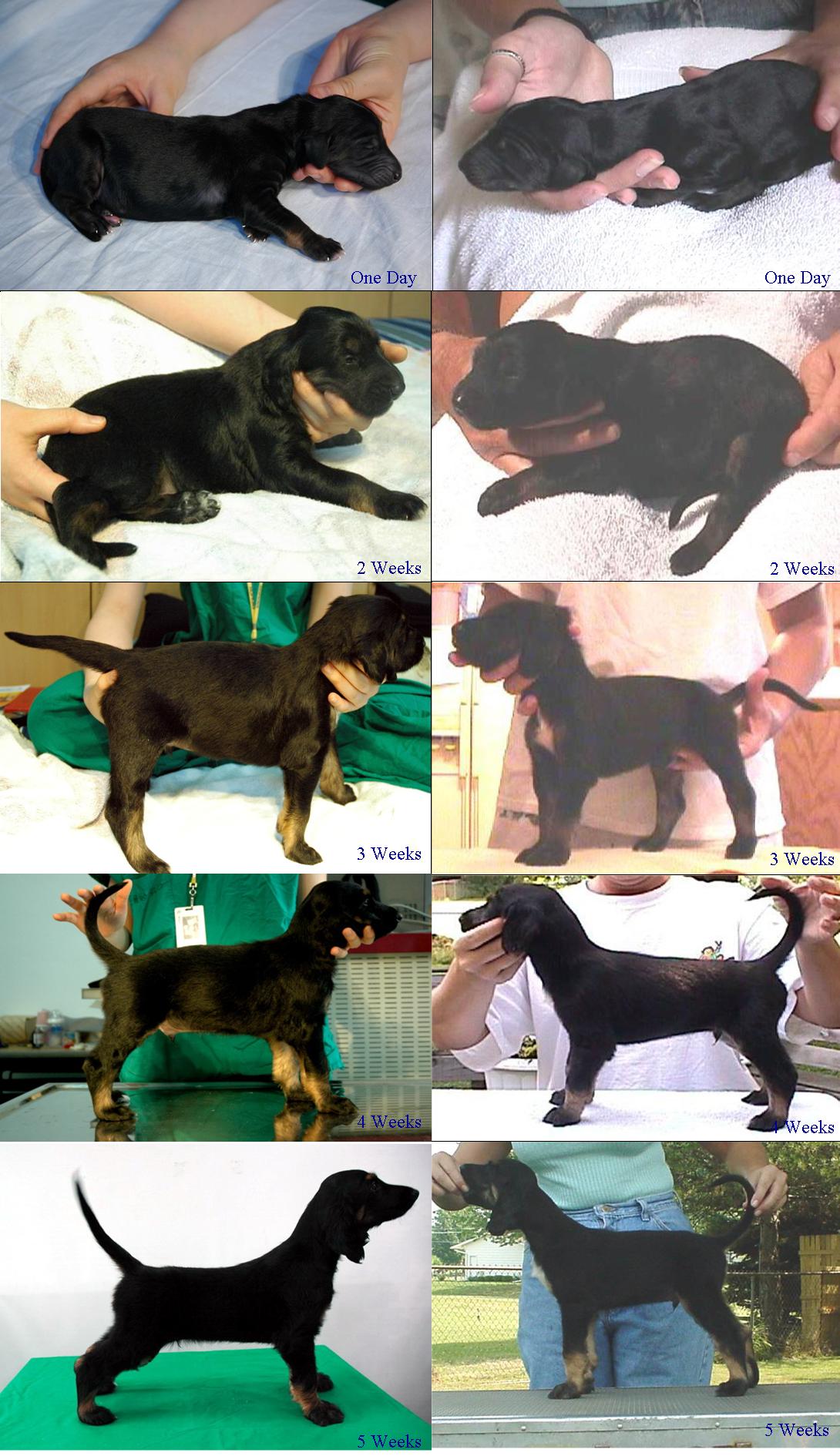
**

**
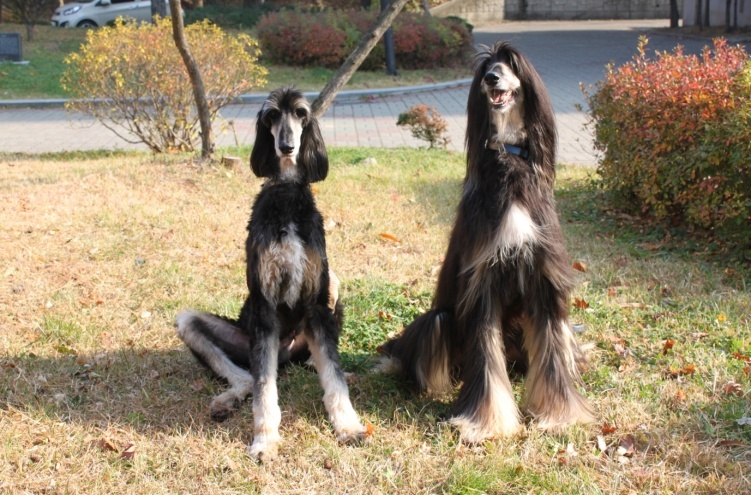
**

**Figure S1.** **Cloned and nuclear donor dogs.** The left is the cloned dog (Snuppy) and the right is the nuclear donor dog (Tai). Snuppy is full grown with no known health problems.


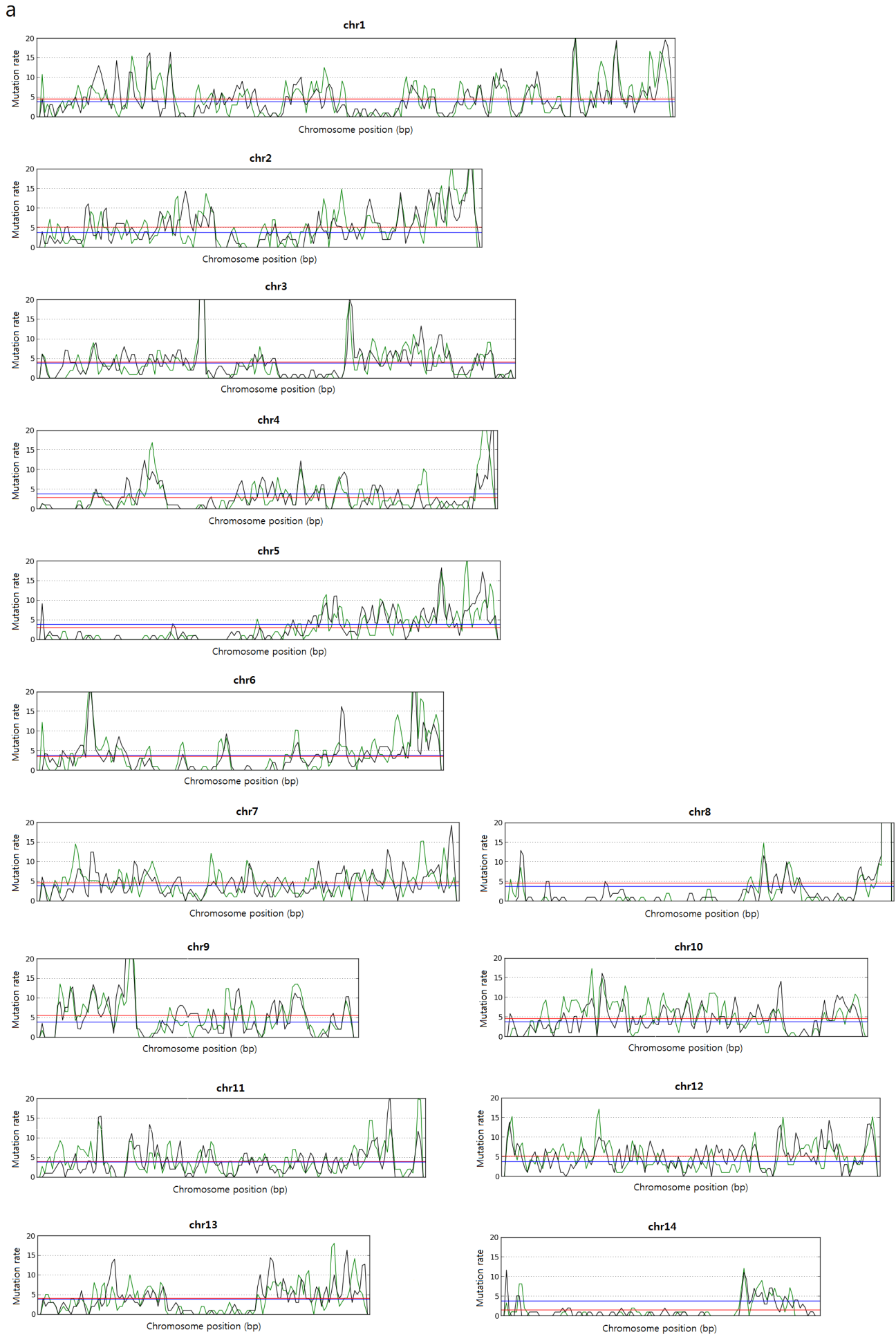


**
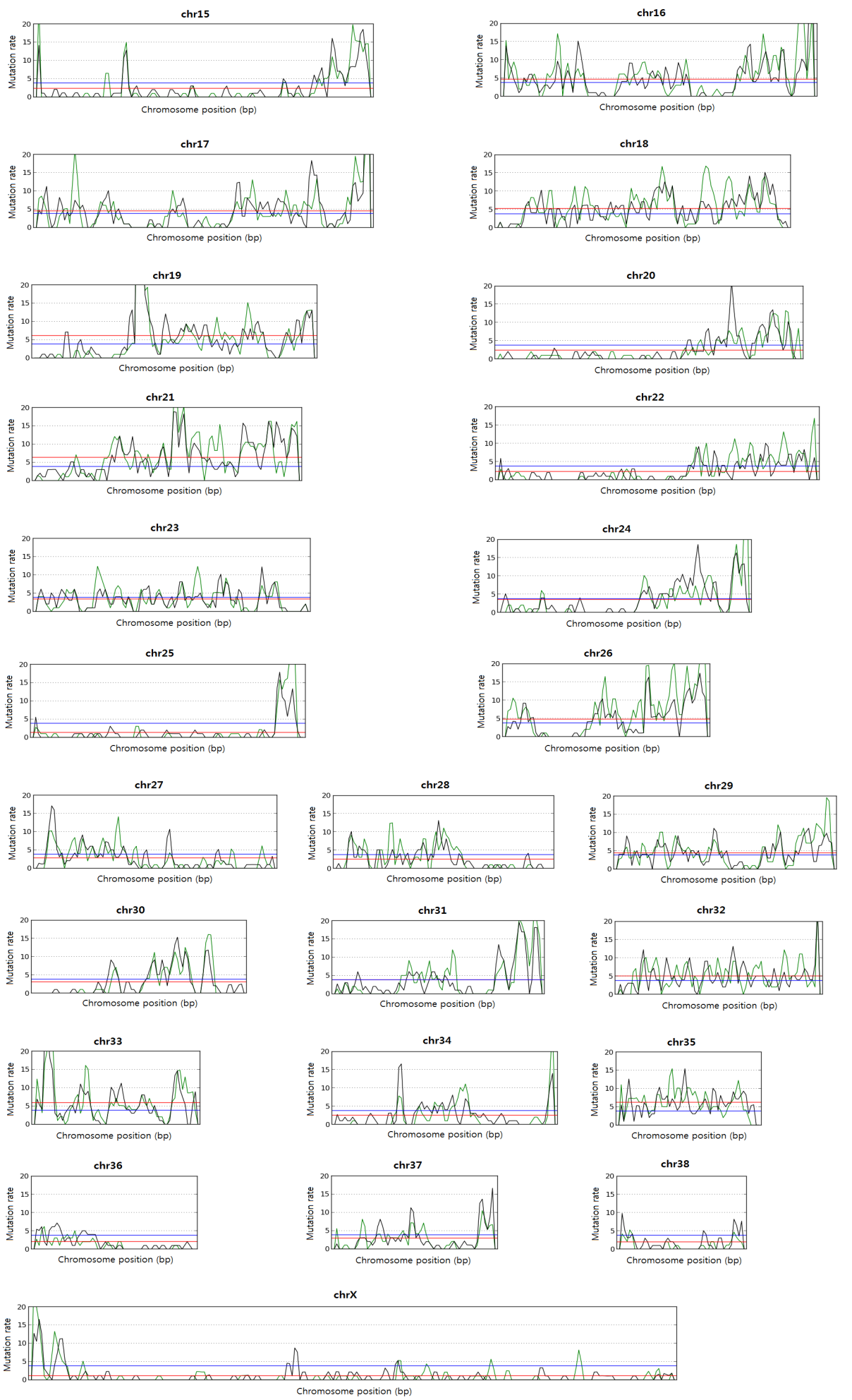
**

**
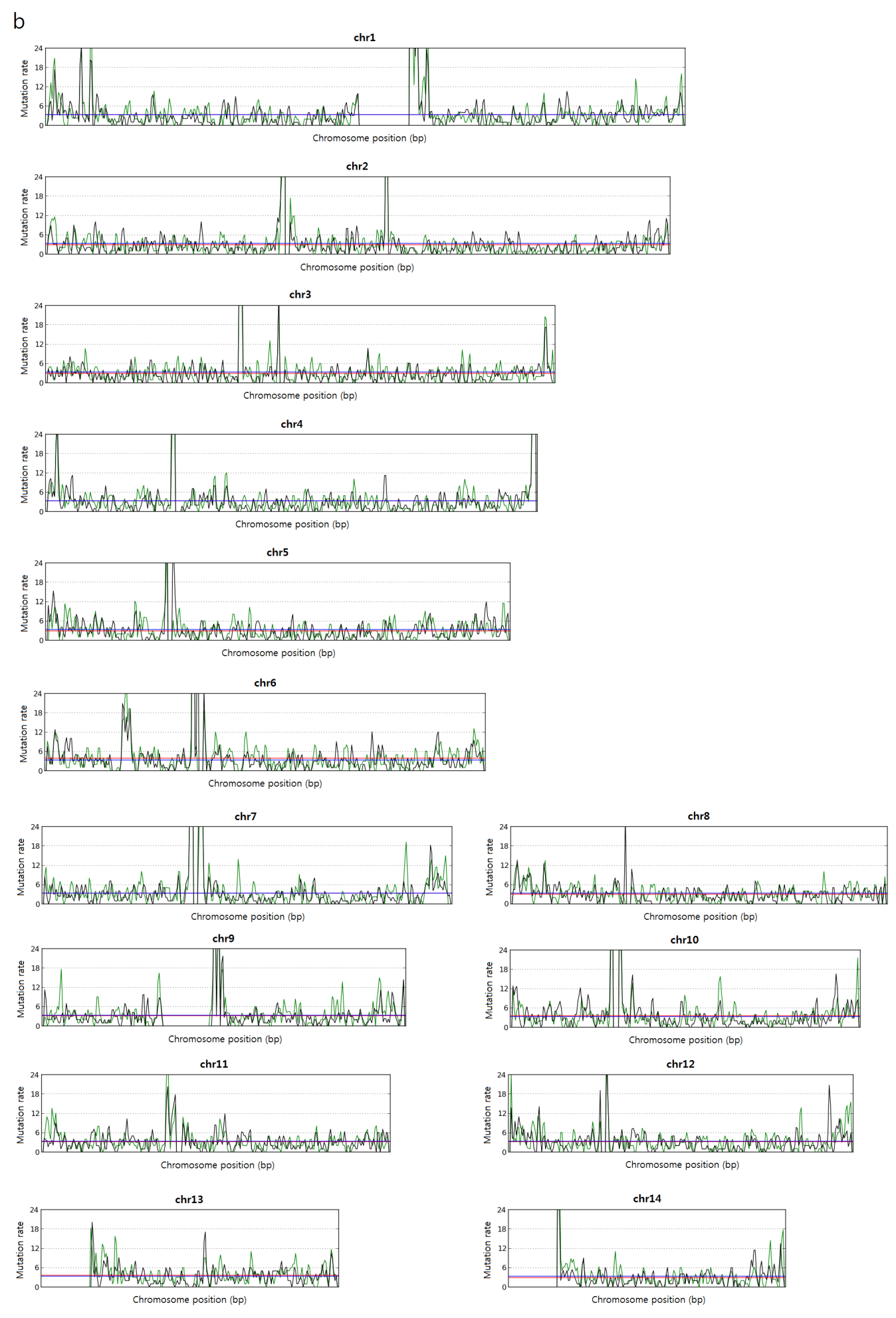
**

**
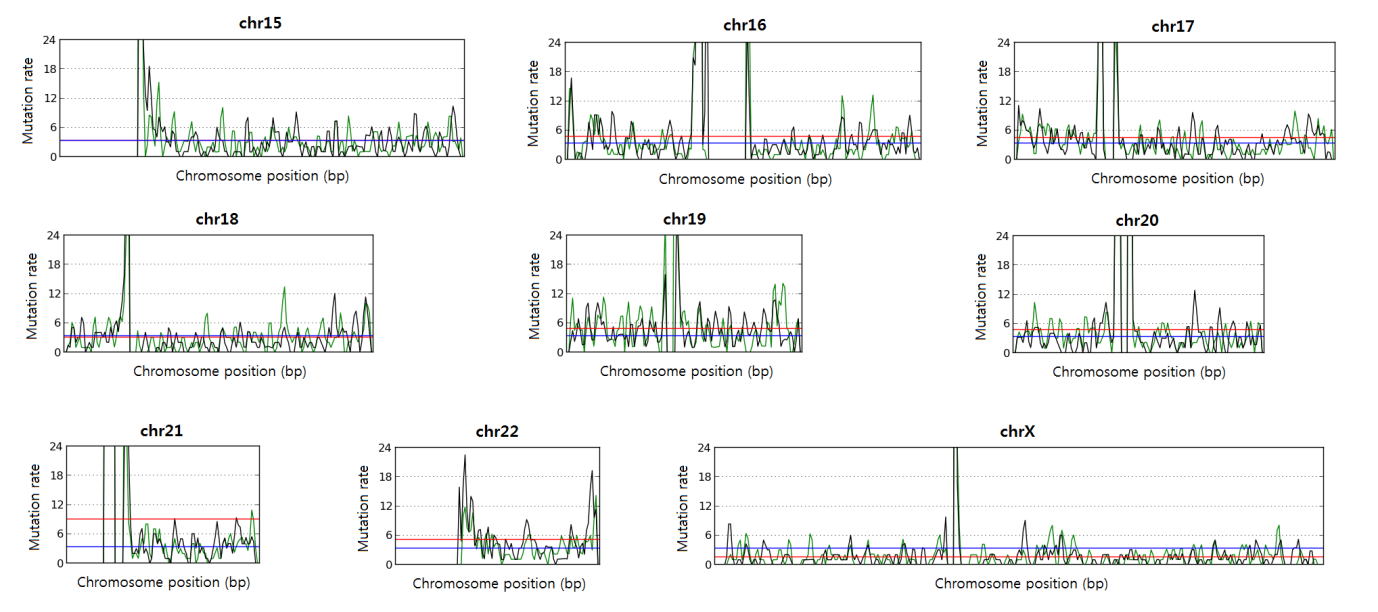
Figure S2.** **Mutation rate distribution of somatic SNV in the dogs and twins.** (**a**) The mutation rates of somatic SNV in the cloned (black) and donor (green) dogs. Red and blue horizontal lines indicate average mutation rate of each chromosome and full length of genome in the cloned dog, respectively. (**b**) The mutation rate of somatic SNV in the monozygotic twins (green: Twin_01, black: Twin_02). Red and blue horizontal lines indicate average mutation rate of each chromosome and full length of genome in the Twin_02, respectively.


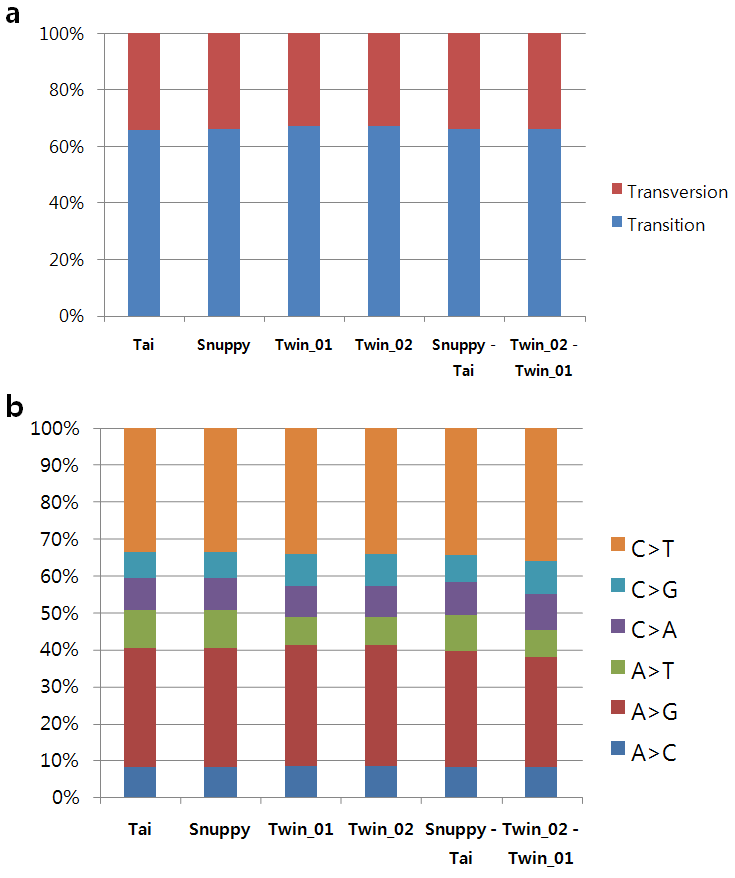


**Figure S3.** **Somatic mutation patterns in the dogs and twins.** (**a**) Transition and transversion ratios. (**b**) Nucleotide substitution ratios.


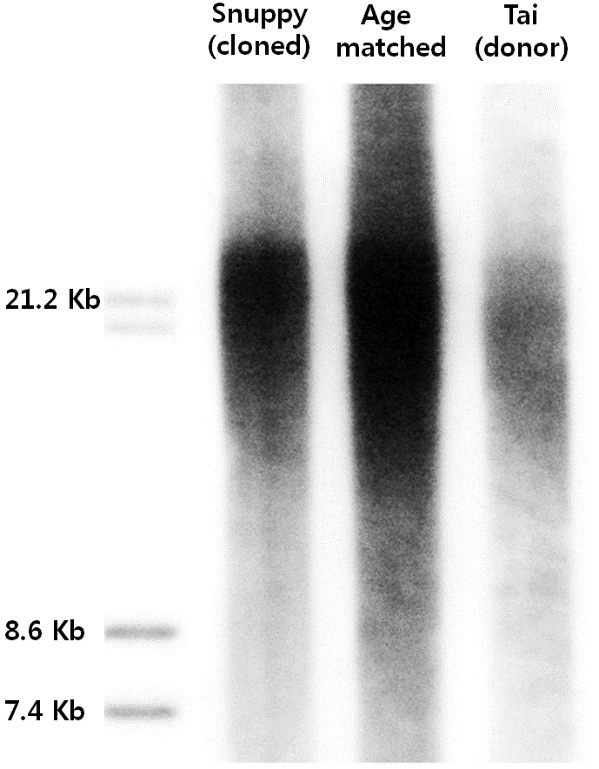


**Figure S4.** **Telomere length estimation using Southern blot.** Telomere length was estimated using the samples of 5 months and 4 months old Snuppy and Tai, respectively. The age matching sample is from the same species (Afghan hound) born by natural breeding.

*Supplementary information tables*

**Table S1.** Sample information.

| Sample | Name | Birth Date | Sex |
| --- | --- | --- | --- |
| Nuclear donor dog | Tai | 2002. 6. 29 | Male |
| Cloned dog | Snuppy | 2005. 4. 24 | Male |
| Human monozygotic twin | Twin_01 | 1990 | Female |
| Human monozygotic twin | Twin_02 | 1990 | Female |

**Table S2.** Statistics regarding filtered sequences.

| Sample | The number of  raw reads | The number of  proper reads | The percentage of  proper reads |
| --- | --- | --- | --- |
| Tai | 793,578,450 | 611,206,572 | 77.02% |
| Snuppy | 718,086,650 | 573,156,177 | 79.82% |
| Twin_01 | 1,073,385,636 | 708,199,074 | 65.98% |
| Twin_02 | 1,088,344,516 | 721,970,140 | 66.34% |

**Table S3.** Statistics regarding mapping sequences.

| Sample | The number of  total reads | The number of  mapped reads | The rate of  mapped reads | The number of  singletone reads | Average mapping depth |
| --- | --- | --- | --- | --- | --- |
| Tai | 611,206,572 | 601,528,947 | 98.42% | 1,550,520 | 22.22 |
| Snuppy | 573,156,177 | 563,128,632 | 98.25% | 1,376,474 | 20.81 |
| Twin_01 | 708,199,074 | 673,833,550 | 95.15% | 3,545,861 | 20.79 |
| Twin_02 | 721,970,140 | 686,476,742 | 95.08% | 3,458,404 | 21.15 |

**Table S4.** Statistics regarding SNVs and indels. The numbers were calculated on the filtered reads mapped to dog (CanFam3.1) and human (hg19) reference genomes.

| Sample | Homozygous SNVs | Heterozygous SNVs | Indels | Total number of variations |
| --- | --- | --- | --- | --- |
| Tai | 2,386,987 | 2,097,294 | 1,133,459 | 5,617,740 |
| Snuppy | 2,375,909 | 2,077,732 | 1,111,928 | 5,565,569 |
| Twin_01 | 1,522,329 | 2,129,710 | 494,223 | 4,146,262 |
| Twin_02 | 1,525,679 | 2,139,964 | 501,360 | 4,167,003 |

**Table S5.** Statistics regarding variations in genomic region.

| Genomic region | Tai | Snuppy | Twin_01 | Twin_02 |
| --- | --- | --- | --- | --- |
| Downstream | 246,060 | 243,522 | 139,368 | 140,498 |
| Intergenic | 3,642,147 | 3,609,219 | 2,464,523 | 2,475,077 |
| Intragenic | 85,062 | 84,297 | 56,934 | 57,259 |
| Upstream | 2,233 | 2,229 | 1,566 | 1,587 |
| 3’ UTR | 18,143 | 17,918 | 19,825 | 19,947 |
| Exon | 26,486 | 26,451 | 22,247 | 22,528 |
| Intron | 1,594,876 | 1,579,221 | 1,440,273 | 1,448,577 |
| 5’ UTR | 2,733 | 2,712 | 1,526 | 1,530 |
| Total | 5,617,740 | 5,565,569 | 4,146,262 | 4,167,003 |

**Table S6.** Statistics regarding somatic SNVs and indels.

| Genomic region | Snuppy - Tai | | Twin_02 - Twin_01 | |
| --- | --- | --- | --- | --- |
|  | SNVs | Indels | SNVs | Indels |
| Downstream | 498 | 264 | 308 | 106 |
| Intergenic | 5,558 | 4,476 | 5,734 | 2,070 |
| Intragenic | 135 | 115 | 134 | 51 |
| Upstream | 4 | 2 | 2 | 1 |
| 3’ UTR | 25 | 17 | 35 | 17 |
| Exon | 58 | 8 | 66 | 3 |
| Intron | 2,254 | 1,989 | 2,828 | 1,260 |
| 5’ UTR | 2 | 1 | 2 | 1 |
| Total | 8,534 | 6,872 | 9,129 | 3,509 |

**Table S7. Statistics regarding somatic mutation rates.**

| Sample | Genome Size | Sufficiently Covered Region | | Sufficiently Covered CDS Region | |
| --- | --- | --- | --- | --- | --- |
|  |  | Size | Mutation Rate (# of SNVs / Mbase) | Size | Mutation Rate (# of SNVs / Mbase) |
|  |  |  |  |  |  |
| Tai | 2,327,650,711 | 2,243,616,364 | 3.84 | 29,712,759 | 1.95 |
| Snuppy |  |  | 3.77 |  | 2.32 |
| Twin_01 | 3,095,693,983 | 2,714,751,451 | 3.36 | 28,309,564 | 1.77 |
| Twin_02 |  |  | 3.57 |  | 1.98 |

**Table S8.** Somatic nsSNVs list in the cloned dog and twin.

| Species | Chromosome | Position | Ref base | Alt base | Genotype | Amino acid change | Gene accession | Gene name | PolyPhen2 |
| --- | --- | --- | --- | --- | --- | --- | --- | --- | --- |
| Cloned dog | 10 | 212284 | G | A | Hetero | P182S | ENSCAFG00000000069 | DNAJC14 | benign |
| Cloned dog | 17 | 61096690 | C | T | Hetero | P120L | ENSCAFG00000030636 | Unknown | unknown |
| Cloned dog | 26 | 6820284 | C | A | Hetero | E1204D | ENSCAFG00000007837 | KNTC1 | benign |
| Cloned dog | 2 | 73660109 | G | A | Hetero | G37R | ENSCAFG00000012496 | ZNF683 | benign |
| Cloned dog | 4 | 25292848 | G | A | Hetero | E1093K | ENSCAFG00000015357 | KAT6B | benign |
| Cloned dog | 7 | 66496811 | A | G | Hetero | K811E | ENSCAFG00000018249 | ESCO1 | probably damaging |
| Twin | 12 | 11420563 | G | T | Hetero | P207Q | NM_006249.4 | PRB3 | benign |
| Twin | 16 | 19475099 | C | A | Hetero | A413D | NM_001261841.1 | TMC5 | benign |
| Twin | 1 | 223177974 | G | A | Hetero | V1079M | NM_032890.3 | DISP1 | benign |
| Twin | 20 | 50407502 | A | C | Hetero | L507R | NM_020436.3 | SALL4 | benign |
| Twin | 6 | 44310854 | G | A | Hetero | G8R | NM_145026.3 | SPATS1 | probably damaging |
| Twin | 9 | 139927660 | G | A | Hetero | D49N | NM_207511.1 | C9orf139 | probably damaging |

**Table S9.** Somatic copy number variation (CNV) list. Only CNVs with log2 copy ratio > ± 0.2 were used.

| Sample | Chr | Length  of CNV | Start | End | Case read | Control read | log2.copyRatio | log10.pvalue |
| --- | --- | --- | --- | --- | --- | --- | --- | --- |
| Snuppy | chrMT | 16,100 | 1 | 16,100 | 201,734 | 163,786 | 0.3956 | -1477.91 |
| Snuppy | chrMT | 300 | 16,101 | 16,400 | 398 | 797 | -0.9069 | -15.46 |
| Snuppy | chrMT | 300 | 16,401 | 16,700 | 2,416 | 2,075 | 0.3144 | -3.42 |
| Twin | chr1 | 800 | 91,852,801 | 91,853,600 | 5,719 | 4,399 | 0.4133 | -36.64 |
| Twin | chr8 | 200 | 70,602,301 | 70,602,500 | 1,110 | 575 | 0.9837 | -31.65 |

**Table S10.** Somatic structural variation (SV) results. CTX is inter-chromosomal translocation; DEL is deletion; ITX is intra-chromosomal translocation; INS is insertion; INV is inversion.

| Type | Somatic | | Individual | | | |
| --- | --- | --- | --- | --- | --- | --- |
|  | Snuppy - Tai | Twin_02 - Twin_01 | Tai | Snuppy | Twin_01 | Twin_02 |
| CTX | 1 | 2 | 99 | 70 | 69 | 73 |
| DEL | 0 | 26 | 585 | 513 | 567 | 573 |
| ITX | 1 | 14 | 28 | 26 | 48 | 46 |
| INS | 10 | 346 | 123 | 108 | 639 | 839 |
| INV | 0 | 6 | 68 | 61 | 36 | 36 |
| Total | 12 | 394 | 903 | 778 | 1359 | 1,569 |

**Table S11.** Somatic structural variation list in the cloned dog. ITX is intra-chromosomal translocation and CTX is inter-chromosomal translocation.

| Chr1 | Chr1  position | Chr2 | Chr2  position | Type | Length of  affected region | BreakDancer  score | # of supporting reads | Gene name | Genic region |
| --- | --- | --- | --- | --- | --- | --- | --- | --- | --- |
| chr3 | 37612114 | chr3 | 37613901 | ITX | 1608 | 99 | 11 | - | - |
| chr6 | 59811640 | chr6 | 59811807 | insertion | -115 | 99 | 10 | - | - |
| chr6 | 69048542 | chr6 | 69048863 | insertion | -125 | 99 | 24 | FAM73A | intron |
| chr10 | 208750 | chr27 | 5936077 | CTX | N/A | 99 | 13 | Unknown | exon |
| chr11 | 17761007 | chr11 | 17761290 | insertion | -107 | 99 | 11 | - | - |
| chr14 | 45867902 | chr14 | 45867991 | insertion | -106 | 99 | 11 | - | - |
| chr18 | 53974227 | chr18 | 53974420 | insertion | -125 | 99 | 10 | - | - |
| chr19 | 16689257 | chr19 | 16689310 | insertion | -141 | 99 | 11 | - | - |
| chr21 | 40731610 | chr21 | 40731924 | insertion | -125 | 99 | 10 | HPS5 | intron |
| chr22 | 34431472 | chr22 | 34431612 | insertion | -99 | 99 | 10 | - | - |
| chr36 | 21150466 | chr36 | 21150477 | insertion | -134 | 99 | 11 | AGPS | intron |
| chrX | 2642797 | chrX | 2642797 | insertion | -152 | 99 | 11 | - | - |

**Table S12.** Relative telomere lengths of Tai and Snuppy.

| # of repeat (TTAGGG) | donor (Tai) | | | cloned (Snuppy) | | | Log_10_(cloned/donor) |
| --- | --- | --- | --- | --- | --- | --- | --- |
|  | Total telomere read pairs | Total  read  pairs | Relative telomere read pairs | Total telomere  read pairs | Total  read  pairs | Relative telomere read pairs |  |
| 1 | 30,062,797 | 396,789,225 | 0.07577 | 27,301,250 | 359,043,325 | 0.07604 | 0.00157 |
| 2 | 284,017 | 396,789,225 | 0.00072 | 267,252 | 359,043,325 | 0.00074 | 0.01699 |
| 3 | 140,452 | 396,789,225 | 0.00035 | 141,540 | 359,043,325 | 0.00039 | 0.04676 |
| 4 | 107,788 | 396,789,225 | 0.00027 | 110,922 | 359,043,325 | 0.00031 | 0.05586 |
| 5 | 99,650 | 396,789,225 | 0.00025 | 103,056 | 359,043,325 | 0.00029 | 0.05801 |
| 6 | 95,481 | 396,789,225 | 0.00024 | 99,160 | 359,043,325 | 0.00028 | 0.05983 |
